# Elevation of Circulating miR-210 Participates in the Occurrence and Development of Type 2 Diabetes Mellitus and Its Complications

**DOI:** 10.1155/2022/9611509

**Published:** 2022-11-23

**Authors:** Xi Chen, Feng Tian, Zhilian Sun, Guoqing Zeng, Ping Tang

**Affiliations:** ^1^Department of General Practice, Shenzhen Luohu People's Hospital, The 3rd Affiliated Hospital of Shenzhen University, Shenzhen, Guangdong, China; ^2^Department of Health Care, The Shunde Affiliated Hospital of Southern Medical University, Guangzhou, Guangdong, China; ^3^Department of Endocrinology and Metabolism, Shenzhen Luohu People's Hospital, The 3rd Affiliated Hospital of Shenzhen University, Shenzhen, Guangdong, China

## Abstract

**Objective:**

Circulating miRNAs are acclaimed biomarkers to predict the occurrence and progression of type 2 diabetes mellitus (T2DM). This study is aimed at analyzing the correlation of circulating miR-210 level and obesity-associated T2DM and then investigating the underlying mechanism of circulating miR-210 in T2DM.

**Methods:**

Totally, 137 serum samples from patients with T2DM were collected; meanwhile, the demographic, general, and clinical hematological characteristics, disease history, and dietary patterns were recorded. The miR-210 level in exosomes from serum was detected by qRT-PCR. Then, the correlations of BMI or miR-210 level with patients' clinical characteristics were analyzed. Furthermore, the miR-210 level was detected in T2DM related various cells under high glucose condition. Meanwhile, the expression of carbohydrate responsive element binding protein (ChREBP) and hypoxia-inducible factor 1*α* (HIF-1*α*) was measured by western blotting.

**Results:**

The miR-210 level in exosomes from serum was obviously elevated in the BMI > 24 group compared with the BMI ≤ 24 group. Higher BMI was correlated with abnormal lipid metabolism and impaired liver function as well as higher miR-210 level. Notably, higher miR-210 level was also correlated with abnormal lipid metabolism, disease history, and dietary patterns. In addition, compared with normal cells, high glucose increased the miR-210 level in exosomes from cell culture supernatants as well as cells in HUVEC, VSMC, RAW 264.7, 3 T3-L1, SMC, and Beta-TC-6 cells, while it reduced the expression of ChREBP and HIF-1*α*.

**Conclusions:**

Circulating miR-210 level was closely correlated with obesity-associated T2DM. Furthermore, higher miR-210 level might be implicated in the occurrence and development of T2DM and its complications.

## 1. Introduction

As a continuous metabolic disease, type 2 diabetes mellitus (T2DM) is caused by the deficiency of insulin secretion and function, accounting for larger than 90% of all DM cases [1, 2]. It is characterized by chronic elevated blood glucose as well as the metabolic disorder of carbohydrate and lipoprotein [3]. Due to inappropriate or delayed treatment, T2DM can cause multisystem damage, thereby developing serious complications, such as retinopathy, nephropathy, neuropathy, and cardiovascular disorders, which impair the quality of life, shorten life span, and increase mortality [1, 4]. Currently, the basic therapy for T2DM is to lower blood glucose by increasing insulin levels via oral drugs that can elevating insulin secretion as well as direct insulin administration [5, 6]. However, the potential adverse drug reactions and complex complications restrict the effectiveness of current treatments [5, 6]. Therefore, exploring the underlying mechanism of T2DM is an urgent need of the effective treatment of T2DM.

MicroRNAs (miRNAs), consisting of approximately 22 nucleotides, are a large type of noncoding small RNA molecules that suppress the expression of target gene through mRNA degradation mediated by the RNA-induced silencing complex in the posttranscriptional level [7]. MicroRNAs possess tissue and developmental stage-specific expression and exert vital roles in mediating several cellular processes, such as cell differentiation, proliferation, and apoptosis, thus substantially involved in the pathogenesis of distinct human disorders [8]. Accumulated evidences have demonstrated that miRNAs dysregulation is involved in occurrence of T2DM and progression of devastating complications [9, 10]. Nowadays, a large number of circulating miRNAs are regarded as promising diagnostic biomarkers and therapeutic targets for T2DM through regulating glucose metabolism, inflammatory response, endothelial dysfunction, and platelet reactivity [11]. Notably, T2DM has been reported to be involved in the occurrence of sleep breathing disorders and is considered as a risk factor for nocturnal hypoxia [12]. Multiple miRNAs have been proved to participate in the critical process of hypoxic response [13, 14]. Specifically, as a hypoxia-related miRNA, miR-210 exists in a broad range of cells and is well reported in various diseases with hypoxic components [15]. Zhang et al. found that miR-210 levels are associated with inflammatory burden [16], while T2DM is also related with increased levels of inflammatory molecules [17]. Inflammatory cytokines [18] and other inflammation markers, such as C-reactive protein [19], serum uric acid [20], monocyte/lymphocyte ratio [21], and uric acid/HDL cholesterol ratio [22] are associated with diabetic complications in diabetic population. Therefore, whether miR-210 affects T2DM and its complications through inflammation is one of the issues concerned in this study. Moreover, recent studies have focused on the role of miR-210 in T2DM, which reveal that the abnormal expression of miR-210 is implicated in the progression of T2DM with retinopathy and coronary artery disease [23, 24]. However, the mechanism of circulating miR-210 in obesity-associated T2DM is still not fully revealed.

In the present study, we first analyzed the correlation of obesity and miR-210 with clinical characteristics of patients with T2DM. Furthermore, we explored the miR-210 level in T2DM related various cells, including endothelial cells, vascular smooth muscle cells (VSMC), macrophages, embryonic fibroblasts (preadipocytes), cardiomyocytes, skeletal muscle cells (SMC), and pancreatic *β* cells under high glucose condition, which might be able to explain the roles of miR-210 in obesity-associated T2DM.

## 2. Materials and Methods

### 2.1. Clinical Samples Collection

Totally, one hundred and thirty-seven serum samples from patients who newly were diagnosed as type 2 diabetes mellitus (T2DM) without treatments at the 3rd Affiliated Hospital of Shenzhen University from January 2020 to June 2021 were included in this study, consisting of 88 males and 49 females aged 22-74 years. Meanwhile, the demographic and general characteristics of T2DM patients, including gender, age, height, weight, body mass index (BMI), head circumference, neck circumference, waist circumference, hip circumference, visceral fat, and subcutaneous fat, were recorded. In addition, the clinical hematological characteristics were collected, including diagnosis time, fasting blood-glucose (FBG), postprandial blood glucose (PBG), fasting insulin (FINS), postprandial insulin (PINS), fasting and postprandial C-peptide, glycated hemoglobin (HbAlc), hemoglobin (HGB), red blood count (RBC), white blood cell (WBC), hematocrit value, mean corpuscular volume (MCV), mean corpuscular hemoglobin (MCH), mean platelet volume (MPV), alanine aminotransferase (ALT), aspartate aminotransferase (AST), alkaline phosphatase (ALP), gamma-glutamyl transpeptidase (GGT), albumin (ALB), blood urea nitrogen (BUN), creatinine (CR), uric acid (UA), triglyceride (TG), total cholesterol (TC), high-density lipoprotein (HDL), and low density lipoprotein (LDL). Moreover, investigations of patient's disease history and dietary patterns were performed. Based on BMI, patients with T2DM were divided into two groups: BMI ≤ 24 and BMI > 24. The differences of serum exosomal miR-210 level, the demographic, general, and clinical characteristics between these two groups were compared. Furthermore, according to the median of serum exosomal miR-210 level, patients were divided into the low miR-210 level group and the high miR-210 level group, and the differences of the demographic, general, and clinical hematological characteristics, disease history, and dietary patterns between the two groups were analyzed. For the serum samples and clinical data used in this study, prior patient consent and approval were obtained from the Ethics Committee of the 3rd Affiliated Hospital of Shenzhen University Hospital.

### 2.2. Cell Culture and Treatments

Human umbilical vein endothelial cells (HUVEC), VSMC, mouse mononuclear macrophages (RAW 264.7), mouse embryonic fibroblasts (preadipocytes, 3 T3-L1), mouse cardiomyocytes (HL-1), mouse SMC, and mouse insulinoma pancreatic *β* cells (Beta-TC-6) were purchased and recovered in complete DMEM medium (Thermo, China) before use and cultured with 5% carbon dioxide at 37°C. All these cells were cultured in DMEM medium containing exosome-depleted FBS for 24 h, and then stimulated with or without high dose of glucose (30 mM), namely, high glucose group (HG) and normal group for 3 h.

### 2.3. Exosome Isolation

Exosomes from serum and cell culture supernatants were isolated by ExoQuick solution (System Biosciences, LLC., USA) as described in the manufacturer's protocol. In brief, serum or cell culture supernatants were incubated with ExoQuick exosome precipitation reagent overnight at 4°C, and then the exosome pellet was obtained by centrifugation at 1,500 g for 30 and 5 minutes, respectively.

### 2.4. Quantitative RT-PCR

The exosomes and treated cells were harvested to obtain total RNA samples by the TAKARA TRIzol Reagent. Then, complementary DNA samples were prepared from the isolated RNA based on the Bestar™ qPCR RT kit (DBI). The following quantitative PCR was finished using the BestarTM qPCR MasterMix (DBI) on fluorescent quantitative PCR instrument (Mx3000P, Agilent Stratagene). U6 was used as the internal standards for miR-210 quantitation. Sequences of primers were all presented in Table S1.

### 2.5. Western Blotting Assay

The treated cells were lysed using lysis buffer (Beyotime, China) on ice to extract proteins. Then, protein samples underwent resolving on PAGE gel and transferring to PVDF, followed by blocking the membrane and reacting with carbohydrate responsive element binding protein (ChREBP), hypoxia-inducible factor 1*α* (HIF-1*α*) or GAPDH primary antibody (1 : 1000, Abcam) at room temperature for 3 h. Second antibody (1 : 3000, Abcam) was then reacted with the membrane, and the expression of these proteins was observed using enhanced chemiluminescence (Millipore, USA).

### 2.6. Statistical Analysis

Quantitative data were analyzed using the SPSS 20.0 software. Quantitative and qualitative data were expressed as mean ± standard deviation (SD) or percentage, respectively. Skewness-Kurtosis normality analysis was applied to the study variables. The differences between two groups were evaluated by *t* test. Pearson correlation analysis was used to analyze the correlations of BMI with miR-210 level, the demographic, general, and clinical characteristics as well as the correlations of the miR-210 level with the demographic, general, and clinical characteristics. A *P* value of <0.05 was set as the threshold.

## 3. Results

### 3.1. The Correlation of BMI with Clinical Characteristics and the Serum Exosomal miR-210 Level in Patients with T2DM

**Figure 1:**
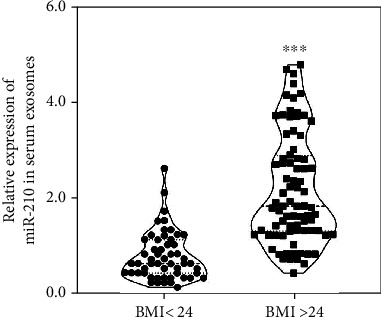
The miR-210 level in exosomes from serum in patients with type 2 diabetes mellitus. A total of 134 patients with type two diabetes mellitus were divided into two groups according the body mass index. There were 54 patients in BMI < 24.0 group and 80 patients in BMI > 24.0 group. The exosomal miR-210 levels from serum were detected by qRT-PCR. ^∗∗∗^*P* < 0.001.

A total of 137 patients with T2DM included in this study, including BMI ≤ 24 (*n* = 57) and BMI > 24 (*n* = 80) groups. The miR-210 level in exosomes from serum was obviously elevated in the BMI > 24 group compared with the BMI ≤ 24 group (Figure 1). In addition, the demographic and general characteristics were compared between BMI ≤ 24 and BMI > 24 groups. The results found higher weight, visceral fat, and subcutaneous fat in the BMI > 24 group than those in the BMI ≤ 24 group, while other characteristics, including gender, age, height, head circumference, neck circumference, waist circumference, hip circumference, showed no significant differences between the two groups (Table 1). Moreover, patients in the BMI > 24 group showed slightly lower RBC as well as higher TC and LDL levels compared with the BMI ≤ 24 group, while there were no significant differences in diagnosis time, FBG, PBG, FINS, PINS, fasting and postprandial C-peptide, HbAlc, HGB, WBC, hematocrit value, MCV, MCH, MPV, ALT, AST, ALP, GGT, ALB, BUN, CR, UA, TG, and HDL between the two groups (Table 2). Pearson correlation analysis also revealed that higher BMI was correlated with higher weight, visceral fat, subcutaneous fat, AST level, ALB level, TG level, TC level, and LDL level (*P* < 0.05, Tables 1 and 2).

### 3.2. The Correlation of Clinical Characteristics and the miR-210 Level in Patients with T2DM

We compared the T2DM patients' characteristics between the low and high miR-210 level groups (Table 3). The results revealed that patients in the high miR-210 level group showed higher weight, visceral fat, subcutaneous fat, BMI, TG, TC, and LDL levels than those in the low miR-210 level group (Table 3). Pearson correlation analysis also revealed that higher miR-210 level was correlated with higher weight, visceral fat, subcutaneous fat, BMI, TG level, TC level, and LDL level (*P* < 0.05, Table 3). We then analyzed the correlation of disease history and dietary patterns with the miR-210 level, and we found that the higher percentages of overeating within 3 months, hypertension, and hyperlipidemia as well as the lower percentages of diabetic pedipathy, coronary heart disease of immediate family, cerebral stroke of immediate family, hypertension of immediate family, and average intakes of fresh vegetable and soy products were correlated with the higher miR-210 level (Tables 4 and 5).

### 3.3. The miR-210 Level in High Glucose-Induced Various Cells

**Figure 2:**
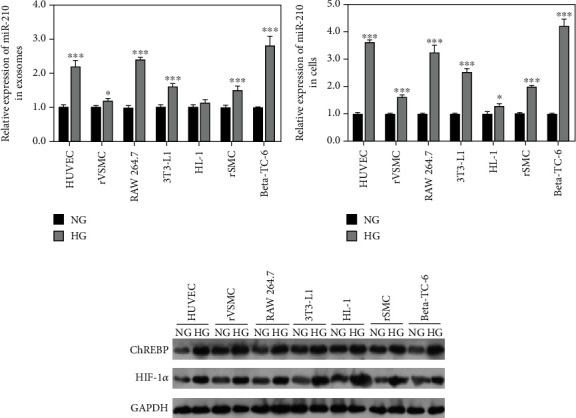
The exosomal miR-210 levels from cell culture supernatants and various cells after high glucose exposure. (a) The exosomal miR-210 level from cell culture supernatants of human umbilical vein endothelial cells (HUVEC), vascular smooth muscle cells (VSMC), mouse mononuclear macrophages (RAW 264.7), mouse embryonic fibroblasts (3 T3-L1), mouse cardiomyocytes (HL-1), mouse skeletal muscle cells (SMC), and mouse insulinoma pancreatic *β* cells (Beta-TC-6) after high glucose exposure by qRT-PCR. (b) The miR-210 level of various cells after high glucose exposure by qRT-PCR. (c) The protein levels of carbohydrate responsive element binding protein (ChREBP), hypoxia-inducible factor 1*α* (HIF-1*α*) in various cells after high glucose exposure by western blotting. ^∗^P < 0.05, ^∗∗∗^*P* < 0.001*vs.* normal group (NG).

We further detected the miR-210 level in various cells after high glucose exposure. The results found that compared with normal cells, exosomes from cell culture supernatants expressed obviously higher miR-210 level in high glucose-induced HUVEC, VSMC, RAW 264.7, 3 T3-L1, SMC, and Beta-TC-6 cells (Figure 2(a)). Similarly, the miR-210 level in high glucose-induced HUVEC, VSMC, RAW 264.7, 3 T3-L1, HL-1, SMC, and Beta-TC-6 cell was remarkably elevated compared to normal cells (Figure 2(b)). Furthermore, we found that the expression of ChREBP and HIF-1*α* which might be transcriptional regulator of miR-210 was decreased in the high glucose-induced various cells in comparison to normal cells (Figure 2(c)).

## 4. Discussion

Increasing evidences have demonstrated that circulating miRNAs are acclaimed biomarkers due to high stability and noninvasive property to predict the occurrence and progression of various diseases, including cancers, metabolic diseases, and inflammatory diseases [25]. miR-210 is known as hypoxia-inducible miRNA to participate in hypoxic response in a variety of diseases [26]. Previous study has proved the overexpression of miR-210 in patients with T2DM [27]. This study aimed to further investigate the influences of miR-210 on T2DM related various cells, thereby evaluating its clinical diagnostic and therapeutic value in T2DM.

Obesity is thought to be a trigger factor for T2DM [28, 29]. It has been indicated that excessive obesity in childhood and adolescence is considered a risk factor for developing T2DM in young adults and young adults [28]. BMI is a common indicator to measure obesity and health, and BMI > 24 is considered as overweight. Thus, in this study, patients with T2DM were divided into two groups according BMI. We found that T2DM patients with BMI > 24 showed higher weight, visceral fat, and subcutaneous fat compared with patients with BMI ≤ 24. In addition, we compared the clinical hematological characteristics between the two groups, and the results revealed that T2DM patients with higher BMI showed slightly lower RBC as well as higher TC and LDL levels. Moreover, we found that higher BMI was correlated with higher weight, visceral fat, subcutaneous fat, and the increased levels AST, ALB, TG, TC, and LDL. It is well known that AST and ALB are key indicators for liver function, and fat consisting of TC, TG, and LDL is used to evaluate the lipid metabolism. Obesity induces abnormal lipid metabolism, and a large number of lipid components into the liver further lead to impaired liver function [30, 31], which well explains our clinical results.

miRNAs have been widely reported to exist in exosomes and are involved in various cellular biological processes by regulating the functions of neighboring or distant cells [32]. A previous study has identified several miRNAs in exosomes from adipose tissue macrophages that medicate glucose homeostasis and insulin sensitivity *in vivo* and *in vitro* [33]. Notably, miR-210 is reported to be upregulated in exosomes from adipose tissue macrophages [33]. Thus, in this study, we detected the miR-210 level of exosomes from serum in patients with T2DM, and the results found that miR-210 level was significantly higher in the BMI > 24 group compared with the BMI ≤ 24 group. In addition, we found that higher miR-210 level was correlated with higher weight, visceral fat, and subcutaneous fat as well as increased BMI, TG, TC, and LDL levels, which suggested that miR-210 overexpression was closely associated with obesity in patients with T2DM. We then analyzed the correlation of disease history and dietary patterns with the miR-210 level and found that the higher miR-210 level was related to the increased percentages of overeating within 3 months, hypertension, and hyperlipidemia as well as the reduced percentages of diabetic pedipathy, coronary heart disease of immediate family, cerebral stroke of immediate family, hypertension of immediate family, and average intakes of fresh vegetable and soy products. All these results indicated the correlation of obesity-associated T2DM with high miR-210 level. To further investigate the mechanism of miR-210 in obesity-associated T2DM, T2DM related various cells, including HUVEC, VSMC, RAW 264.7, 3 T3-L1, SMC, and Beta-TC-6 cells, were exposed to high glucose condition. All these cells are implicated in the occurrence and development of T2DM and its complications. Accumulated evidence has shown that endothelial cell dysfunction such as cell apoptosis induced by inflammatory response oxidative stress is predisposing factor of diabetic vascular lesions [34, 35]. Meanwhile, excessive proliferation of VSMC also contributes to the occurrence of cardiovascular complications of T2DM through inducing the thickening of the artery walls and formation of atherosclerotic plaques [36]. It has also been reported that T2DM can induce macrophages to produce inflammatory factors, which results in accelerated inflammatory response [37]. In addition to cardiovascular diseases and inflammatory reaction, T2DM is also reported to develop skeletal myopathy [38]. More importantly, the reduction and dysfunction of pancreatic *β* cells is the underlying causes of T2DM [39]. Our results showed that miR-210 was highly expressed in high glucose-induced HUVEC, VSMC, RAW 264.7, 3 T3-L1, SMC, and Beta-TC-6 cells both in exosomes from cell culture supernatants and cells, which suggested that miR-210 overexpression is closely associated with the occurrence and progression of T2DM and its complications by regulating distinct type of cells. These results are consistent with previous studies that report the overexpression of miR-210 in diabetes complicated with impaired wound healing, cardiac angiogenesis, and retinopathy [23, 40, 41]. Moreover, HIF-1*α* has been proved to be a target gene of miR-210 [42], and HIF-1*α* is one of miR-210's transcriptional regulators [43]. During T2DM, HIF-1 signaling has been reported to participate in *β* cell dysfunction, thereby resulting in the occurrence of diabetic complications through regulating angiogenesis and apoptosis [44]. In addition, ChREBP is a critical regulatory factor for glycolysis and fat formation and is involved in the deregulation of HIF-1 signaling under high glucose condition [45]. Consistently, this study revealed that high glucose inhibited the expression of HIF-1*α* and ChREBP. Therefore, we speculated that the abnormal expression of miR-210 during the occurrence and progression of T2DM and its complications might be regulated by HIF-1 signaling. However, it is widely known that the level of noncoding RNA in serum is dynamically changed. Because the blood samples of patients at different time periods of the day were not collected, it is difficult to observe the dynamic change of miR-210 level in this study. Meanwhile, the lack of cell experiments to verify the role and mechanism of miR-210 in T2DM and its complications is also one of the limitations of this study.

## 5. Conclusion

In conclusion, data form our study revealed that circulating high miR-210 level was closely correlated with obesity-associated T2DM. Furthermore, circulating miR-210 might be implicated in the occurrence and development of T2DM and its complications, and the expression of miR-210 might be regulated by HIF-1*α* and ChREBP.

## Figures and Tables

**Table 1:** The differences and correlation of demographic, general characteristics, and miR-210 level between BMI ≤ 24 and BMI > 24 groups in patients with type 2 diabetes mellitus.

	BMI ≤ 24 (*n* = 57)	BMI > 24 (*n* = 80)	*t* test (*P*)	Pearson *r*	*P*
Gender			0.102	—	—
Male	32	56			
Female	25	24			
Age (year)	57.39 ± 9.72	55.46 ± 8.22	0.227	-0.107	0.097
Height (cm)	161.16 ± 9.09	163.53 ± 7.37	0.107	0.143	0.094
Weight (kg)	58.25 ± 7.62	71.37 ± 8.81	<0.001	0.616	<0.001
Head circumference (cm)	55.83 ± 2.53	55.33 ± 2.15	0.226	-0.107	0.214
Neck circumference (cm)	37.28 ± 3.23	36.24 ± 4.81	0.131	-0.122	0.157
Neck circumference (cm)	88.31 ± 9.12	87.60 ± 9.66	0.661	-0.037	0.664
Hip circumference (cm)	95.93 ± 6.24	96.50 ± 6.27	0.597	-0.045	0.598
Visceral fat (cm^2^)	72.17 ± 20.70	96.75 ± 36.20	<0.001	0.369	<0.001
Subcutaneous fat (cm^2^)	141.75 ± 39.05	199.35 ± 56.26	<0.001	0.498	<0.001
Fold change miR-210 level	0.77 ± 0.50	2.16 ± 1.16	<0.001	0.592	<0.001

BMI: body mass index.

**Table 2:** The differences and correlation of clinical characteristics and miR-210 level between BMI ≤ 24 and BMI > 24 groups in patients with type 2 diabetes mellitus.

	*BMI* ≤ 24 (*n* = 57)	*BMI* > 24 (*n* = 80)	*t* test (*P*)	Pearson *r*	*P*
Diagnosis time (month)	88.98 ± 70.89	91.46 ± 76.43	0.845	0.019	0.828
FBG (mmol/L)	7.76 ± 1.83	7.75 ± 2.36	0.989	0.039	0.647
PBG (mmol/L)	11.81 ± 4.73	11.97 ± 4.45	0.837	0.003	0.969
FINS (*μ*IU/mL)	11.49 ± 8.96	10.84 ± 7.01	0.649	-0.007	0.933
PINS (*μ*IU/mL)	44.11 ± 39.48	43.40 ± 28.42	0.907	-0.027	0.752
Fasting C-peptide(*μ*g/L)	2.11 ± 1.03	2.31 ± 1.88	0.445	-0.010	0.909
Postprandial C-peptide (*μ*g/L)	5.87 ± 2.72	6.14 ± 3.48	0.615	-0.069	0.420
HbAlc (%)	0.08 ± 0.02	0.07 ± 0.02	0.942	0.046	0.597
HGB (g/L)	147.75 ± 16.28	143.9 ± 17.25	0.185	-0.142	0.097
RBC (×10^12/L)	5.11 ± 0.56	4.92 ± 0.56	0.049	-0.162	0.058
WBC (×10^9/L)	7.06 ± 1.86	7.10 ± 1.76	0.898	-0.002	0.981
PLT (×10^9/L)	264.86 ± 78.93	263.54 ± 69.83	0.919	0.063	0.462
Hematocrit *value*	9.60 ± 69.08	0.99 ± 4.94	0.07	-0.025	0.773
MCV (fl)	88.53 ± 6.08	88.40 ± 8.70	0.913	-0.064	0.459
MCH (pg)	30.96 ± 14.83	29.55 ± 2.65	0.483	-0.028	0.745
MPV (fl)	10.26 ± 0.92	10.55 ± 1.15	0.097	0.001	0.991
ALT (IU/L)	22.74 ± 12.17	23.65 ± 12.71	0.672	0.031	0.718
AST (IU/L)	21.42 ± 7.46	22.86 ± 10.79	0.357	0.180	0.035
ALP (IU/L)	78.11 ± 25.70	84.78 ± 22.79	0.120	0.121	0.158
GGT (IU/L)	29.72 ± 18.17	34.19 ± 41.67	0.396	0.159	0.063
ALB (g/L)	45.63 ± 2.69	45.73 ± 6.28	0.906	-0.175	0.041
BUN (mmol/L)	5.56 ± 1.32	5.79 ± 2.78	0.525	0.055	0.523
CR (*μ*mol/L)	71.28 ± 22.63	80.85 ± 93.18	0.380	0.036	0.680
UA (*μ*mol/L)	350.60 ± 83.83	349.46 ± 83.08	0.937	0.020	0.813
TG (mmol/L)	2.02 ± 3.78	2.27 ± 2.29	0.665	0.196	0.022
TC (mmol/L)	4.96 ± 1.37	5.67 ± 2.39	0.028	0.335	<0.001
HDL (mmol/L)	1.11 ± 0.28	1.19 ± 0.36	0.156	-0.002	0.979
LDL (mmol/L)	2.74 ± 0.75	3.24 ± 1.20	0.003	0.397	<0.001

BMI: body mass index; FBG: fasting blood-glucose; PBG: postprandial blood glucose; FINS: fasting insulin; PINS: postprandial insulin; HbAlc: glycated hemoglobin; HGB: hemoglobin; RBC: red blood count; WBC: white blood cell; MCV: mean corpuscular volume; MCH: mean corpuscular hemoglobin; MPV: mean platelet volume; ALT: alanine aminotransferase; AST: aspartate aminotransferase; ALP: alkaline phosphatase; GGT: gamma-glutamyl transpeptidase; ALB: albumin; BUN: blood urea nitrogen; CR: creatinine; UA: uric acid; TG: triglyceride; TC: total cholesterol; HDL: high-density lipoprotein; LDL: low density lipoprotein.

**Table 3:** The differences and correlation of demographic, general, and clinical characteristics between high and low miR-210 level groups in patients with type 2 diabetes mellitus.

	L-miR-210 (*n* = 69)	H-miR-210 (*n* = 68)	*t* test (*P*)	Pearson *r*	*P*
Gender			0.102	—	—
Male	32	56			
Female	25	24			
Age (year)	56.52 ± 10.00	56.00 ± 7.68	0.732	-0.022	0.8
Height (cm)	160.98 ± 8.61	164.13 ± 7.46	0.023	0.161	0.061
Weight (kg)	59.91 ± 7.89	71.99 ± 9.38	<0.001	0.684	<0.001
Head circumference (cm)	55.74 ± 2.52	55.32 ± 2.09	0.294	-0.092	0.285
Neck circumference (cm)	37.22 ± 5.09	36.11 ± 3.10	0.126	-0.13	0.129
Neck circumference (cm)	87.96 ± 9.70	87.82 ± 9.18	0.934	-0.024	0.779
Hip circumference (cm)	96.31 ± 6.37	96.20 ± 6.15	0.923	-0.026	0.767
Visceral fat (cm^2^)	73.51 ± 24.71	99.73 ± 35.11	<0.001	0.557	<0.001
Subcutaneous fat (cm^2^)	151.86 ± 48.35	199.25 ± 55.99	<0.001	0.476	<0.001
BMI	23.10 ± 1.80	26.69 ± 2.34	<0.001	0.835	<0.001
Diagnosis time (month)	89.33 ± 80.05	91.54 ± 67.71	0.862	0.075	0.386
FBG (mmol/L)	7.56 ± 1.64	7.95 ± 2.57	0.297	0.046	0.595
PBG (mmol/L)	11.53 ± 4.43	12.28 ± 4.68	0.34	0.044	0.611
FINS (*μ*IU/mL)	11.40 ± 8.76	10.82 ± 6.87	0.668	0.032	0.713
PINS (*μ*IU/mL)	46.58 ± 39.01	40.76 ± 26.33	0.307	-0.013	0.876
Fasting C-peptide(*μ*g/L)	2.15 ± 1.01	2.30 ± 2.00	0.569	-0.063	0.466
Postprandial C-peptide (*μ*g/L)	6.19 ± 2.96	5.86 ± 3.40	0.544	-0.140	0.103
HbAlc (%)	7.35 ± 1.44	7.64 ± 2.19	0.368	0.048	0.575
HGB (g/L)	145.07 ± 16.08	145.94 ± 17.81	0.765	-0.077	0.374
RBC (×10^12/L)	5.05 ± 0.55	4.94 ± 0.58	0.256	-0.122	0.155
WBC (×10^9/L)	7.01 ± 1.97	7.16 ± 1.62	0.629	-0.007	0.932
PLT (×10^9/L)	267.36 ± 78.50	260.76 ± 68.41	0.601	-0.034	0.695
Hematocrit value	0.44 ± 0.04	0.44 ± 0.05	0.781	-0.116	0.177
MCV (fl)	88.17 ± 5.91	88.74 ± 9.19	0.669	-0.043	0.621
MCH (pg)	30.49 ± 13.51	29.78 ± 2.74	0.670	-0.067	0.438
MPV (fl)	10.25 ± 0.91	10.61 ± 1.18	0.050	0.054	0.529
ALT (IU/L)	22.90 ± 12.38	23.65 ± 12.61	0.726	0.058	0.501
AST (IU/L)	21.80 ± 7.45	22.74 ± 11.32	0.567	0.123	0.153
ALP (IU/L)	79.41 ± 24.33	84.63 ± 23.91	0.207	0.079	0.358
GGT (IU/L)	32.32 ± 26.08	32.34 ± 40.53	0.997	0.114	0.184
ALB (g/L)	46.12 ± 4.30	45.25 ± 5.77	0.319	-0.196	0.021
BUN (mmol/L)	5.57 ± 1.47	5.81 ± 2.89	0.539	0.057	0.505
CR (*μ*mol/L)	69.28 ± 23.51	84.57 ± 100.15	0.224	0.034	0.690
UA (*μ*mol/L)	351.03 ± 84.42	348.81 ± 82.32	0.877	0.017	0.846
TG (mmol/L)	1.61 ± 0.94	2.74 ± 4.08	0.030	0.180	0.035
TC (mmol/L)	4.85 ± 1.00	5.91 ± 2.64	0.003	0.350	<0.001
HDL (mmol/L)	1.17 ± 0.40	1.14 ± 0.25	0.629	-0.052	0.545
LDL (mmol/L)	2.68 ± 0.69	3.39 ± 1.25	<0.001	0.389	<0.001

BMI: body mass index; FBG: fasting blood-glucose; PBG: postprandial blood glucose; FINS: fasting insulin; PINS: postprandial insulin; HbAlc: glycated hemoglobin; HGB: hemoglobin; RBC: red blood count; WBC: white blood cell; MCV: mean corpuscular volume; MCH: mean corpuscular hemoglobin; MPV: mean platelet volume; ALT: alanine aminotransferase; AST: aspartate aminotransferase; ALP: alkaline phosphatase; GGT: gamma-glutamyl transpeptidase; ALB: albumin; BUN: blood urea nitrogen; CR: creatinine; UA: uric acid; TG: triglyceride; TC: total cholesterol; HDL: high-density lipoprotein; LDL: low density lipoprotein.

**Table 4:** The correlation of disease history with high and low miR-210 level groups in patients with type 2 diabetes mellitus.

	L-miR-210 (*n* = 69)	H-miR-210 (*n* = 68)	Pearson *r*	*P*
Overdrink within 3 months?	No (61)/Yes (8)	No (53)/Yes (15)	2.685	0.101
Overeating within 3 months?	No (64)/Yes (5)	No (55)/Yes (13)	4.229	**0.040**
More urine within 3 months?	No (55)/Yes (14)	No (54)/Yes (14)	0.002	0.965
Infection within 3 months?	No (67)/Yes (2)	No (64)/Yes (4)	0.728	0.393
Hypoglycemia within 3 months?	No (59)/Yes (10)	No (63)/Yes (5)	1.791	0.181
Numbness in hands and feet within 3 months?	No (54)/Yes (15)	No (53)/Yes (15)	0.002	0.964
Physical pain or paresthesia within 3 months?	No (60)/Yes (9)	No (63)/Yes (5)	1.209	0.272
Blurred vision within 3 months?	No (47)/Yes (22)	No (49)/Yes (19)	0.254	0.614
Lower limb edema within 3 months?	No (68)/Yes (1)	No (66)/Yes (2)	0.356	0.551
Intermittent claudication within 3 months?	No (59)/Yes (10)	No (63)/Yes (5)	1.791	0.181
Have you lost weight in the past year?	No change (46)	No change (52)	4.471	0.215
	0 ~ 2.5 kg (14)	0 ~ 2.5 kg (10)		
	2.5 ~ 5.0 kg (7)	2.5 ~ 5.0 kg (2)		
	>5.0 kg(2)	>5.0 kg(4)		
Diabetic pedipathy?	No (59)/Yes (10)	No (66)/Yes (2)	5.718	**0.017**
Do you have a definite diagnosis of hypertension?	No (43)/Yes (26)	No (28)/Yes (40)	6.132	**0.013**
Whether you are or have had hyperlipidemia?	No (48)/Yes (21)	No (30)/Yes (38)	9.045	**0.003**
Whether you have or have had high uric acid?	No (58)/Yes (11)	No (55)/Yes (13)	0.239	0.625
Urine protein?	No (55)/Yes (14)	No (56)//Yes (12)	0.156	0.693
Urine glucose?	No (53)/Yes (16)	No (50)/Yes (18)	0.198	0.657
Coronary heart disease?	No (62)/Yes (7)	No (66)/Yes (2)	2.896	0.089
History of stroke?	No (62)/Yes (7)	No (65)/Yes (3)	1.664	0.197
Thyroid disease?	No (65)/Yes (4)	No (60)/Yes (8)	1.526	0.217
Hepatic disease?	No (57)/Yes (12)	No (53)/Yes (15)	0.472	0.492
Biliary tract disease?	No (61)/Yes (8)	No (63)/Yes (5)	0.717	0.397
Pancreatic disease?	No (67)/Yes (2)	No (66)/Yes (2)	0.000	0.988
Gastrointestinal disease?	No (59)/Yes (10)	No (59)/Yes (9)	0.045	0.831
Urinary system diseases?	No (61)/Yes (8)	No (58)/Yes (10)	0.291	0.590
Respiratory disorders?	No (67)/Yes (2)	No (65)/Yes (3)	0.223	0.637
Trauma history?	No (59)/Yes (10)	No (59)/Yes (9)	0.045	0.831
Surgical treatment?	No (51)/Yes (18)	No (47)/Yes (21)	0.387	0.534
Insulin-related antibody GAD	No (67)/Yes (2)	No (67)/Yes (1)	0.326	0.568
Insulin-related antibody ICA	No (69)/Yes (0)	No (68)/Yes (0)	—	—
Insulin-related antibody IAA	No (69)/Yes (0)	No (68)/Yes (0)	—	—
Have you ever used insulin since you were diagnosed with diabetes?	No (55)/Yes (14)	No (57)/Yes (11)	0.388	0.533
Immediate family members with diabetes?	No (21)/Yes (48)	No (28)/Yes (40)	1.720	0.190
Immediate family members with coronary heart disease?	No (49)/Yes (20)	No (59)/Yes (9)	5.091	0.024
Immediate family members with stroke	No (40)/Yes (29)	No (54)/Yes (14)	7.311	0.007
Immediate family members with hypertension?	No (22)/Yes (47)	No (33)/Yes (35)	3.949	0.047
Immediate family members with obesity?	No (52)/Yes (17)	No (53)/Yes (15)	0.127	0.721
Immediate family members with hyperlipemia?	No (57)/Yes (12)	No (54)/Yes (14)	0.228	0.633

**Table 5:** The correlation of dietary patterns with high and low miR-210 level groups in patients with type 2 diabetes mellitus.

	L-miR-210 (*n* = 69)	H-miR-210 (*n* = 68)	Pearson	*P*
Frequency of smoking (per week)	Never (54)	Never (54)	0.306	0.858
	1 ~ 6 times (5)	1 ~ 6 times (6)		
	≥7 times (10)	≥7 times (8)		

Frequency of drinking (per week)	Never (35)	Never (38)	0.425	0.808
	1 ~ 6 times (24)	1 ~ 6 times (22)		
	≥7 times (10)	≥7 times (8)		

Average intake of fresh vegetable (per day)	<200 g (12)	<200 g (24)	8.889	**0.015**
	200~400 g (35)	200~400 g (28)		
	400~600 g (18)	400~600 g (16)		
	>600 g (4)	>600 g (0)		

Average intake of fresh fruit (per day)	<200 g (39)	<200 g (36)	3.417	0.332
	200~400 g (26)	200~400 g 27)		
	400~600 g (2)	400~600 g (5)		
	>600 g (2)	>600 g (0)		

Frequency of fish consumption (per week)	≤1 times (25)	≤1 times (27)	0.176	0.675
	≥2 times (44)	≥2 times (41)		

Average intake of soy products (per day)	<100 g (22)	<100 g (36)	9.656	**0.010**
	100~250 g (32)	100~250 g (21)		
	250~400 g (11)	250~400 g (11)		
	>400 g (4)	>400 g (0)		

Are you still working?	No (36)	No (30)	0.89	0.345
	Yes (33)	Yes (38)		

Sleep status (past 7 days)	Fell bad (28)	Fell bad (20)	1.877	0.171
	Fell good (41)	Fell good (48)		

## Data Availability

The data used to support the findings of this study are available from the corresponding author upon request.

## Additional Points

*Highlights*. (i) Higher BMI was correlated with higher miR-210 level in patients with T2DM. (ii) Higher miR-210 level was correlated with abnormal lipid metabolism, disease history, and dietary patterns. (ii) High glucose increased the miR-210 level in T2DM related various cells

## Supplementary Materials

Supplementary Materials Table S1: specific primers for qPCR assay.

## References

[1] Zheng Y., Ley S. H., Hu F. B. (2018). Global aetiology and epidemiology of type 2 diabetes mellitus and its complications. *Nature Reviews Endocrinology*.

[2] Blair M. (2016). Diabetes mellitus review. *Urologic Nursing*.

[3] Galicia-Garcia U., Benito-Vicente A., Jebari S. (2020). Pathophysiology of type 2 diabetes mellitus. *International Journal of Molecular Sciences*.

[4] Cole J. B., Florez J. C. (2020). Genetics of diabetes mellitus and diabetes complications. *Nature Reviews Nephrology*.

[5] Xu L., Li Y., Dai Y., Peng J. (2018). Natural products for the treatment of type 2 diabetes mellitus: pharmacology and mechanisms. *Pharmacological Research*.

[6] Overbeek J. A., Heintjes E. M., Prieto-Alhambra D. (2017). Type 2 diabetes mellitus treatment patterns across europe: a population-based multi-database study. *Clinical Therapeutics*.

[7] Hammond S. M. (2015). An overview of microRNAs. *Advanced Drug Delivery Reviews*.

[8] Chen X., Xie D., Zhao Q., You Z. H. (2019). MicroRNAs and complex diseases: from experimental results to computational models. *Briefings in Bioinformatics*.

[9] Regazzi R. (2018). MicroRNAs as therapeutic targets for the treatment of diabetes mellitus and its complications. *Expert Opinion on Therapeutic Targets*.

[10] Tiwari J., Gupta G., de Jesus Andreoli Pinto T. (2018). Role of microRNAs (miRNAs) in the pathophysiology of diabetes mellitus. *Panminerva Medica*.

[11] Pordzik J., Jakubik D., Jarosz-Popek J. (2019). Significance of circulating microRNAs in diabetes mellitus type 2 and platelet reactivity: bioinformatic analysis and review. *Cardiovascular Diabetology*.

[12] Khandelwal D., Dutta D., Chittawar S., Kalra S. (2017). Sleep disorders in type 2 diabetes. *Indian journal of endocrinology and metabolism*.

[13] Gupta A., Sugadev R., Sharma Y., Ahmad Y., Khurana P. (2018). Role of miRNAs in hypoxia-related disorders. *Journal of Biosciences*.

[14] Serocki M., Bartoszewska S., Janaszak-Jasiecka A., Ochocka R. J., Collawn J. F., Bartoszewski R. (2018). miRNAs regulate the hif switch during hypoxia: a novel therapeutic target. *Angiogenesis*.

[15] Bavelloni A., Ramazzotti G., Poli A. (2017). miRNA-210: a current overview. *Anticancer Research*.

[16] Zhang J., He J., Luo Y., Liu, Fan (2020). miR-210 regulates the inflammation of otitis media with effusion by inhibiting the expression of hypoxia-inducible factor (hif)-1a. *Biochemical and Biophysical Research Communications*.

[17] Reinehr T. (2019). Inflammatory markers in children and adolescents with type 2 diabetes mellitus. *Clinica Chimica Acta*.

[18] Kocak M. Z., Aktas G., Atak B. M. (2020). Is neuregulin-4 a predictive marker of microvascular complications in type 2 diabetes mellitus?. *European Journal of Clinical Investigation*.

[19] Bilgin S., Kurtkulagi O., Atak Tel B. M. (2021). Does C-reactive protein to serum albumin ratio correlate with diabetic nephropathy in patients with type 2 diabetes mellitus? The care time study. *Primary Care Diabetes*.

[20] Kocak M. Z., Aktas G., Duman T. T., Atak B. M., Savli H. (2019). Is uric acid elevation a random finding or a causative agent of diabetic nephropathy?. *Revista da Associação Médica Brasileira*.

[21] Kocak M. Z., Aktas G., Duman T. T. (2020). Monocyte lymphocyte ratio as a predictor of diabetic kidney injury in type 2 diabetes mellitus; the madkid study. *Journal of Diabetes and Metabolic Disorders*.

[22] Aktas G., Kocak M. Z., Bilgin S., Atak B. M., Duman T. T., Kurtkulagi O. (2020). Uric acid to hdl cholesterol ratio is a strong predictor of diabetic control in men with type 2 diabetes mellitus. *The Aging Male*.

[23] Yin C., Lin X., Sun Y., Ji X. (2020). Dysregulation of miR-210 is involved in the development of diabetic retinopathy and serves a regulatory role in retinal vascular endothelial cell proliferation. *European Journal of Medical Research*.

[24] Amr K., Abdelmawgoud H., Ali Z., Shehata S., Raslan H. M. (2018). Potential value of circulating microRNA-126 and microRNA-210 as biomarkers for type 2 diabetes with coronary artery disease. *British Journal of Biomedical Science*.

[25] Condrat C. E., Thompson D. C., Barbu M. G. (2020). miRNAs as biomarkers in disease: latest findings regarding their role in diagnosis and prognosis. *Cell*.

[26] Chan Y. C., Banerjee J., Choi S. Y., Sen C. K. (2012). miR-210: the master hypoxamiR. *Microcirculation*.

[27] Li X., Jia Z., Zhao X., Xu M., Chen M. (2020). Expression of miR-210 in the peripheral blood of patients with newly diagnosed type 2 diabetes mellitus and its effect on the number and function of endothelial progenitor cells. *Microvascular Research*.

[28] Malone J. I., Hansen B. C. (2019). Does obesity cause type 2 diabetes mellitus (t2dm)? Or is it the opposite?. *Pediatric Diabetes*.

[29] Perng W., Oken E., Dabelea D. (2019). Developmental overnutrition and obesity and type 2 diabetes in offspring. *Diabetologia*.

[30] Nuttall F. Q. (2015). Body mass index: obesity, BMI, and health: a critical review. *Nutrition Today*.

[31] Adab P., Pallan M., Whincup P. H. (2018). *Is Bmi the Best Measure of Obesity?*.

[32] Zhang J., Li S., Li L. (2015). Exosome and exosomal microRNA: trafficking, sorting, and function. *Genomics, Proteomics & Bioinformatics*.

[33] Ying W., Riopel M., Bandyopadhyay G. (2017). Adipose Tissue Macrophage-Derived Exosomal miRNAs Can Modulate _In Vivo_ and _In Vitro_ Insulin Sensitivity. *Cell*.

[34] Kaur R., Kaur M., Singh J. (2018). Endothelial dysfunction and platelet hyperactivity in type 2 diabetes mellitus: molecular insights and therapeutic strategies. *Cardiovascular Diabetology*.

[35] Katakami N. (2018). Mechanism of development of atherosclerosis and cardiovascular disease in diabetes mellitus. *Journal of Atherosclerosis and Thrombosis*.

[36] Vallurupalli S., Mehta J. L. (2017). Vascular remodeling in diabetes mellitus. *Mechanisms of Vascular Defects in Diabetes Mellitus*.

[37] Ying W., Fu W., Lee Y. S., Olefsky J. M. (2020). The role of macrophages in obesity-associated islet inflammation and *β*-cell abnormalities. *Nature Reviews Endocrinology*.

[38] D’Souza D. M., Al-Sajee D., Hawke T. J. (2013). Diabetic myopathy: impact of diabetes mellitus on skeletal muscle progenitor cells. *Frontiers in Physiology*.

[39] Eizirik D. L., Pasquali L., Cnop M. (2020). Pancreatic *β*-cells in type 1 and type 2 diabetes mellitus: different pathways to failure. *Nature Reviews Endocrinology*.

[40] Dallas A., Trotsyuk A., Ilves H. (2019). Acceleration of diabetic wound healing with PHD2-and miR-210-targeting oligonucleotides. *Tissue Engineering Part A*.

[41] Naderi R., Mohaddes G., Mohammadi M. (2019). The effect of garlic and voluntary exercise on cardiac angiogenesis in diabetes: the role of miR-126 and miR-210. *Arquivos Brasileiros de Cardiologia*.

[42] Liu L.-L., Li D., He Y.-L. (2017). miR-210 protects renal cell against hypoxia-induced apoptosis by targeting hif-1 alpha. *Molecular Medicine*.

[43] Xu W., Liu S., Li N. (2021). Quercetin antagonizes glucose fluctuation induced renal injury by inhibiting aerobic glycolysis via HIF-1*α*/miR-210/ISCU/FeS pathway. *Frontiers in Medicine*.

[44] Gunton J. E. (2020). Hypoxia inducible factors and diabetes. *The Journal of clinical investigation*.

[45] Owczarek A., Gieczewska K. B., Jarzyna R., Frydzinska Z., Winiarska K. (2021). Transcription factor chrebp mediates high glucose-evoked increase in hif-1*α* content in epithelial cells of renal proximal tubules. *International Journal of Molecular Sciences*.

