# Supplementary material for: Elevation of Circulating miR-210 Participates in the Occurrence and Development of Type 2 Diabetes Mellitus and Its Complications
Source: J Diabetes Res. 2022 Nov 23;2022:9611509. doi: 10.1155/2022/9611509 (PMC9711992; doi:10.1155/2022/9611509)
Supplement: Supplementary Materials — Table S1: specific primers for qPCR assay. [file 9611509.f1.docx]

**Supplementary Material**

**Table S1. Specific primers for qPCR assay**

| **Gene** | **Primer sequence** |
| --- | --- |
| miR-210 | sense primer: 5'-CTGTGCGTGTGACAGCGGCTGA-3' |
|  | antisense primer: 5'-TTGACACGCACACTGTCGCCGA-3' |
| U6 | sense primer: 5'-CTCGCTTCGGCAGCACA-3' |
|  | antisense primer: 5'-AACGCTTCACGAATTTGCGT-3' |
